# Evaluation of Hepatic Tissue Blood Flow Using Xenon Computed Tomography with Fibrosis Progression in Nonalcoholic Fatty Liver Disease: Comparison with Chronic Hepatitis C

**DOI:** 10.3390/ijms15011026

**Published:** 2014-01-14

**Authors:** Ryuta Shigefuku, Hideaki Takahashi, Masaki Kato, Yoshihito Yoshida, Keigo Suetani, Yohei Noguchi, Moriaki Hatsugai, Kazunari Nakahara, Hiroki Ikeda, Minoru Kobayashi, Kotaro Matsunaga, Nobuyuki Matsumoto, Chiaki Okuse, Fumio Itoh, Shiro Maeyama, Shigeru Sase, Michihiro Suzuki

**Affiliations:** 1 Division of Gastroenterology and Hepatology, Department of Internal Medicine, St. Marianna University School of Medicine, Kawasaki 216-8511, Kanagawa, Japan; E-Mails: r2shigefuku@marianna-u.ac.jp (R.S.); masaki0801_3@marianna-u.ac.jp (M.K.); y3yoshida@marianna-u.ac.jp (Y.Y.); k2suetani@marianna-u.ac.jp (K.S.); y2noguchi@marianna-u.ac.jp (Y.N.); m.h@marianna-u.ac.jp (M.H.); nakahara@marianna-u.ac.jp (K.N.); ikedahi@marianna-u.ac.jp (H.I.); m3kobayashi@marianna-u.ac.jp (M.K.); kotarom@marianna-u.ac.jp (K.M.); nobu1020@marianna-u.ac.jp (N.M.); c2okuse@marianna-u.ac.jp (C.O.); fitoh@marianna-u.ac.jp (F.I.); michstmu@marianna-u.ac.jp (M.S.); 2 Shiodome Medical Examination Clinic, Tokyo 105-0013, Japan; E-Mail: shiromaeyama915@msg.biglobe.ne.jp; 3 Anzai Medical Co., Ltd., Tokyo 141-0033, Japan; E-Mail: sase@anzai-med.co.jp; 4 Division of Gastroenterology and Hepatology, Department of Internal Medicine, Kawasaki Municipal Tama Hospital, Kawasaki 214-8525, Kanagawa, Japan; 5 Division of Gastroenterology, Department of Internal Medicine, Sapporo Shirakabadai Hospital, Sapporo 062-0052, Hokkaido, Japan

**Keywords:** nonalcoholic steatohepatitis, chronic hepatitis C, hepatic tissue blood flow, Xe computed tomography

## Abstract

**Aims:**

The present study evaluated the utility of xenon computed tomography (Xe-CT) as a noninvasive diagnostic procedure for the measurement of hepatic tissue blood flow (TBF) in patients with nonalcoholic fatty liver disease (NAFLD) or chronic hepatitis C (CH-C).

**Methods:**

Xe-CT was performed in 93 patients with NAFLD and in 109 patients with CH-C. Subjects were classified into one of three groups, based on fibrosis stage: group 1, no bridging fibrosis; group 2, bridging fibrosis; and group 3, liver cirrhosis. Correlations between hepatic TBFs in each fibrosis stage were examined.

**Results:**

In group 1, portal venous TBF (PVTBF), hepatic arterial (HATBF), and total hepatic TBF (THTBF) were significantly lower in patients with in nonalcoholic steatohepatitis (NASH) than in those with CH-C (*p* < 0.001, *p* < 0.05, *p* < 0.001, respectively). In group 2, PVTBF and THTBF were significantly lower in patients with in NASH than in those with CH-C (*p* < 0.001, *p* < 0.05, respectively). In group 3, hepatic TBFs were not significantly different when comparing patients with NASH and those with CH-C.

**Conclusions:**

PVTBF decreased due to fat infiltration. Therefore, hemodynamic changes occur relatively earlier in NAFLD than in CH-C. Patients with NASH should be monitored carefully for portal hypertensive complications in the early fibrosis stage.

## 1. Introduction

The liver receives a dual blood supply from the portal vein and hepatic artery. These systems use independent mechanisms for adjustment of blood flow. Hepatic TBF has been evaluated using various noninvasive methods, based on advances in imaging modalities, such as ultrasonography (US) [1–4], computed tomography (CT) [5], and magnetic resonance imaging (MRI) [6]. For other methods, such as color Doppler US [1–3], contrast-enhanced US [4], and angiography [7], intravascular hepatic TBF is calculated by measuring flow velocities and vessel diameters of the portal vein and hepatic artery. Polasek *et al.* reported that molecular MRI of liver fibrosis with a collagen-specific probe identifies fibrotic tissue in two rodent models of disease [8,9]. Xe-CT is a form of perfusion imaging that combines xenon gas inhalation with CT to quantify and visualize TBF [10]. As it is a convenient and non-invasive method, Xe-CT has been widely used in neurosurgical practice to evaluate cerebral TBF [10,11]. In patients with chronic liver disease, HTBF generally decreases with disease progression [12].

There are many reports concerning noninvasive testing to distinguish fibrosis stage in patients with chronic liver disease [2,13,14]. Schneider *et al.* described noninvasive assessment of liver steatosis, fibrosis and inflammation in CH-C [13] and reported that, although Duplex-Doppler of the portal and hepatic veins is not a substitute for histologic grading and staging, portal vein undulations can predict liver cirrhosis with considerable accuracy. We previously reported that the PVTBF measured using Xe-CT reflects progression of fibrosis in CH-C and NASH [15–17].

Moreover, in patients with liver cirrhosis, we previously reported that hepatic TBF varied according to the etiology of the disease [18–20]. However, no studies have compared the TBF between NAFLD and CH-C in detail. Therefore, the goal of the present study was to evaluate the utility of Xe-CT as a noninvasive diagnostic procedure for the measurement of hepatic TBF in patients with NAFLD or CH-C, and characterize the difference in the hepatic TBF between patients with initial chronic hepatitis and those with liver cirrhosis.

## 2. Results

### 2.1. Hepatic TBF in NAFLD Patients

**Figure 1.**
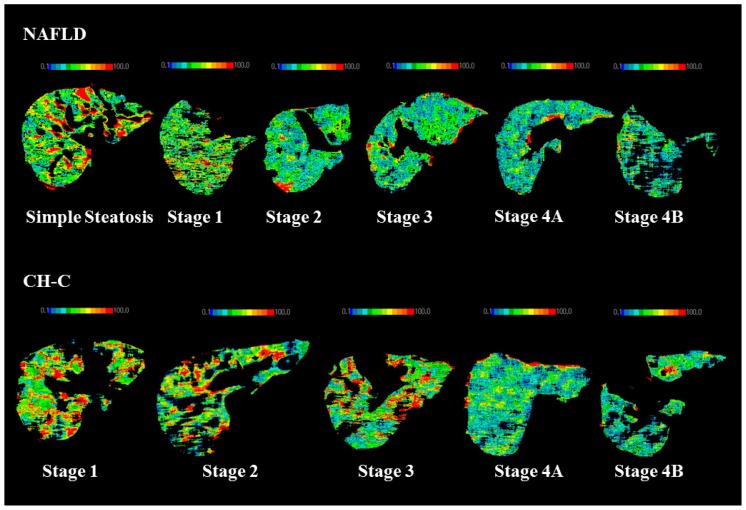
Typical PVTBF maps of NAFLD and CH-C in each fibrosis stage. Measurement of hepatic tissue blood flows (TBFs) and confidence values obtained using xenon computed tomography (Xe-CT). The color in each blood flow map changes from blue to red with increasing blood flow.

The TBF values in NAFLD are shown in Table 1. PVTBF in SS was significantly higher than those in the other stages (*p* < 0.05, *p* < 0.05, *p* < 0.001, *p* < 0.001, *p* < 0.001, respectively). PVTBF was higher in stage 1 and stage 2 than in stage 4B (*p* < 0.01, *p* < 0.05, respectively). PVTBF maps changed from green to blue according to advancing fibrosis stage in NAFLD (Figure 1). HATBF was not significantly different among the fibrosis stages.

THTBF in SS was significantly higher than those in stage 2, stage 3, stage 4A, and stage 4B (*p* < 0.01, *p* < 0.05, *p* < 0.01, *p* < 0.001, respectively). THTBF in stage 1 was significantly higher than that in stage 4B (*p* < 0.05). With fibrosis progression, PVTBF and THTBF decreased significantly (*p* < 0.001, *r* = −0.487, *p* < 0.001, *r* = −0.449, respectively).

### 2.2. Hepatic TBF in CH-C Patients

The TBF values in NAFLD are shown in Table 2. PVTBF in stage 1 was significantly higher than those in the other stages (*p* < 0.01, *p* < 0.01, *p* < 0.001, *p* < 0.001, respectively). PVTBF in stage 2 was significantly higher than that in stage 4B (*p* < 0.01). PVTBF maps changed from green to blue according to advancing fibrosis stage in CH-C (Figure 1). HATBF was not significantly different among the fibrosis stages. THTBF in stage 1 was significantly higher than those in the other stages (*p* <0.01, *p* < 0.01, *p* < 0.001, *p* < 0.001, respectively). With fibrosis progression, PVTBF and THTBF decreased significantly (*p* < 0.001, *r* = −0.550, *p* < 0.001, *r* = −0.408, respectively).

### 2.3. Hepatic TBF in Each Group

**Figure 2.**
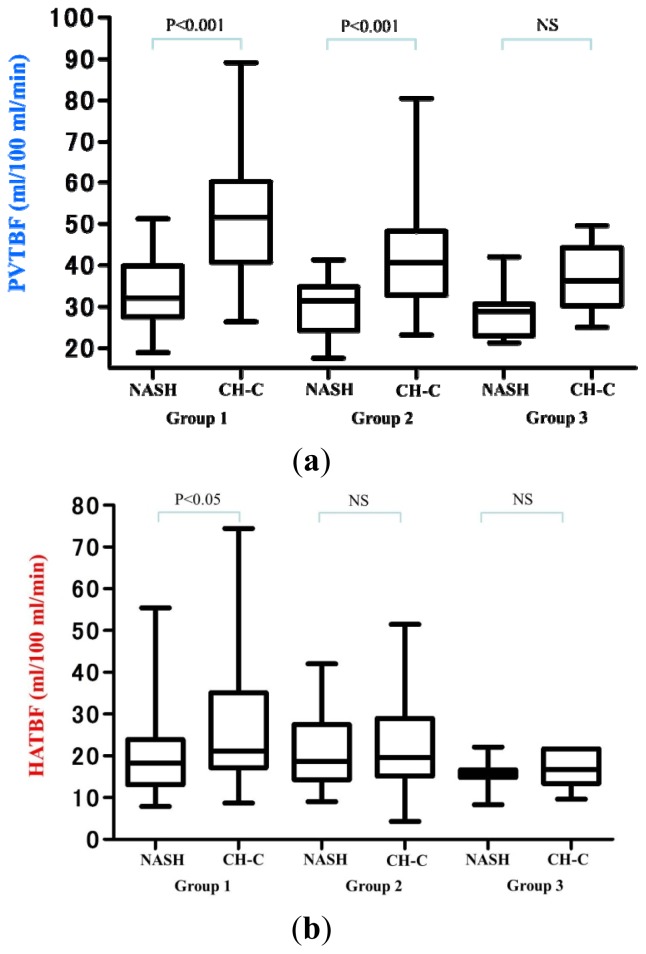

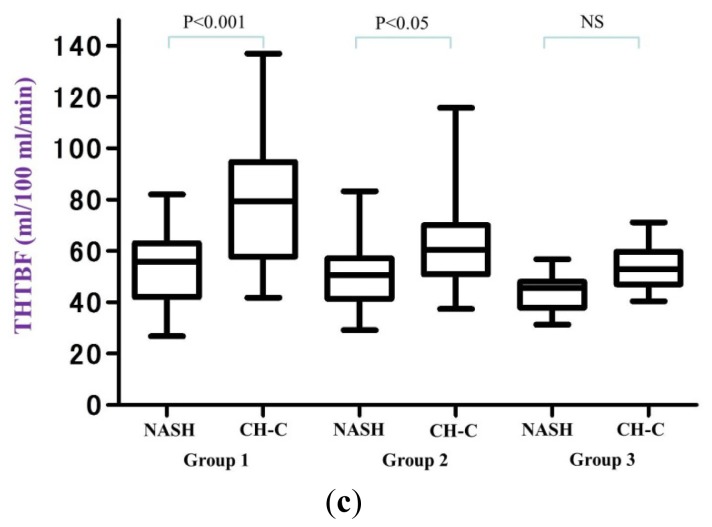
Comparison of hepatic TBF between NASH and CH-C. (**a**) PVTBF; (**b**) HATBF; (**c**) THTBF; NS: not significant.

The TBF values in each group are shown in Table 3 and in Figure 2. In group 1, PVTBF, HATBF and THTBF in NASH were significantly lower than those in CH-C (*p* < 0.001, *p* < 0.05, *p* < 0.001, respectively). In group 2, PVTBF and THTBF in NASH were significantly lower than those in CH-C (*p* < 0.001, *p* < 0.05, respectively). In group 3, hepatic TBFs were not significantly different between NASH and CH-C.

## 3. Discussion

In chronic liver disease, hepatic TBF generally decreases with disease progression [12]. Numerous studies have reported that portal venous flow decreases and a compensatory increase in hepatic arterial flow probably occurs to maintain total HBF with progression of liver disease. Kobayashi *et al.* [16] assessed the usefulness of Xe-CT, as a noninvasive method of quantitatively and visually determining hepatic TBF, and xenon solubility (λ value) simultaneously with TBF, in the evaluation of NASH pathophysiology. We assessed the difference in the hepatic TBF between patients with initial chronic hepatitis and those with liver cirrhosis in NASH and CH-C. Furthermore, we attempted to clarify influence of hepatic TBF on accumulation of fat in the liver by comparison NASH with CH-C. Furthermore, in the present study, we attempt to clarify influence of hepatic TBF on hepatic steatosis in the patients with NASH and CH-C.

**Figure 3.**
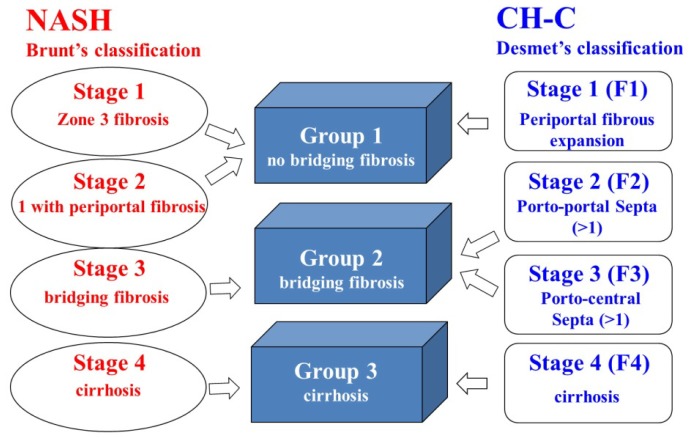
Matching fibrosis stage.

The manner of fibrosis progression in NASH is different from that in CH-C. Initial fibrosis appears periportal area in CH-C. On the other hand, initial fibrosis appears around the area of central vein in NASH. With fibrosis progression, PVTBF decreases in both CH-C and NASH. However, the manner of hepatic TBF in NASH is different from that in CH-C. We definitely recognize the difference between Brunt’s classification and Desmet’s classification. As the manner of progression of liver remodeling differs between NAFLD and CH-C, we cannot directly compare both hepatic TBF. Therefore, to match the fibrotic element, we devised this classification (our original classification) (Figure 3). By satisfying the fibrotic condition using our classification, we make it possible to compare the hepatic TBF between NASH and CH-C. Despite the absence of fibrosis in SS, PVTBP was similar when comparing SS and CH-C stage 2. This suggests that PVTBF and THTBF in NASH were lower than that in CH-C in the earlier fibrosis stage.

Mendes *et al.* investigated the prevalence and noninvasive predictors of portal hypertension in patients with NAFLD [21]. They reported that one hundred of 354 NAFLD patients had portal hypertension at the time of NAFLD diagnosis. Further, of the 204 patients with no or mild fibrosis (stages, 0–2), 12 patients had portal hypertension (6%), meaning that they had a significantly higher grade of steatosis, based on biopsy analysis when compared with the 192 patients without portal hypertension (94%). Therefore, we considered that the hemodynamic changes in NAFLD occurred relatively earlier than those in CH-C.

Alteration in hepatic microcirculation in fatty human donor livers was first observed during organ retrieval before mobilization by Seifalian *et al.* [22] using laser Doppler flowmetry. A significant reduction in hepatic microcirculation in liver donors with steatosis was found when compared with that in normal liver donors [23]. Experimental studies in animal models with fatty liver showed that fatty infiltration, classified as mild (*i.e*., <30%), moderate (*i.e*., 30%–60%), or severe (*i.e*., >60%), reduces hepatic blood flow and microcirculation, and that there was an inverse correlation between the degree of fat infiltration and both total hepatic blood flow and flow in the microcirculation [23]. The severity of steatosis has a greater effect on the microcirculation than on total liver blood flow [24,25]. In addition, Hayashi *et al.* [26] studied alcoholic liver disease and measured hepatic blood flow using a hydrogen clearance method. These investigators reported that hepatic blood flow decreased with fatty change and fibrotic change. With fatty change alone, hepatic blood flow decreased, but with both fatty change and fibrotic change, hepatic blood flow clearly decreased further.

The characteristics of NASH resemble the histopathological findings of alcoholic liver disease; thus, the relationship between fatty change, fibrotic change, and hepatic blood flow, is thought to be similar when comparing patients with NASH and patients with alcoholic disease. In previous studies using Xe-CT, we compared hepatic TBF in patients with alcoholic cirrhosis, NASH cirrhosis, and cirrhosis due to HCV. Although patients with early cirrhosis were examined, the hepatic TBF was lower in patients with alcoholic cirrhosis and NASH cirrhosis when compared with that in patients with cirrhosis due to HCV [17,18]. This result reflects the finding that, in addition to fibrotic change, fatty change and hepatocyte ballooning physically compress the sinusoids and impair the parenchymal microcirculation; as a result, hepatic blood flow is further decreased.

The diffusion to the organization of the Xe gas does not have a difference. We describe the influence of Xe diffusion and hepatic TBF in the liver in the case of severe liver steatosis. The λ value is expressed by the following equation: *k* = *C*h/*C*v, where *C*h is the Xe solubility coefficient in liver and *C*v is the Xe solubility coefficient in blood. Normal values are *C*h = 0.1 and *C*v = 0.14; thus, the theoretical LV for normal liver is 0.1/0.14 = 0.714. The Xe solubility coefficient of water is 0.59, and the Xe solubility coefficient of adipose tissue is 9.3 [27,28]. Thus, the λ value increases with increased fat deposition in liver tissue. The λ value is an independent coefficient of blood flow and is higher in tissues with greater Xe solubility. Therefore liver steatosis couldn’t influence Xe diffusion in the liver thereby affecting the calculated indices of hepatic TBF based on Fick’s principle.

Fat content decreases with fibrosis stage progression in NASH (*i.e*., “burned-out NASH”). Our result showed that PVTBF in NASH was significantly lower than that in CH-C in group 1. However, PVTBF in NASH was not significantly different from that in CH-C in group 3. This phenomenon suggests that the influence of steatosis leading to a decrease is PVTBF was weaker in group 3 than that in the other groups.

PVTBF decreases as result of steatosis and fibrosis. On the other hand, HATBF is relatively maintained despite steatosis and fibrosis. There compensatory mechanism called the “hepatic arterial buffer response” maintains THTBF by increasing HATBF in response to decreasing PVTBF [29,30]. In the present study, the hepatic arterial buffer response (HABR) appeared in CH-C in stage 4B and appeared in NASH in stage 3. In fact, HABR appeared at an earlier fibrosis stage in NASH than in CH-C. This phenomenon suggests that PVTBF was lower in NASH than in CH-C at an earlier fibrosis stage.

Hepatic TBF in SS was lower than that in initial CH-C patients. This suggests that SS can result from microcirculatory dysfunction [31] due to steatosis, dysfunction of the microvessels, and the effects of vasoactivators (e.g., angiotensin and inducible NO synthase (iNOS)), hormones (e.g., endothelin), and cytokines (e.g., tumor necrosis factor-α) [32–34]. Thus, in addition histopathological findings, such as fibrosis and steatosis, cytokines and metabolic factors, such as insulin resistance, are involved in decreased hepatic TBF in NASH.

There are some demerits of Xe-CT. We can’t obtain the exact result in the patients with severe lung disease because Xe gas is taken up by lung via respiratory tract. In addition, Xe-CT has the problem of X-ray exposure. On the other hand there are also many merits of Xe-CT. We can objectively and repeatedly measure hepatic TBF with reproducibility using Xe-CT. We safely performed a Xe-CT for patients with chronic kidney disease because there are no complications associated with contrast agent, such as allergic reactions and radiocontrast-induced renal failure. Eventually, in the present study all patients had no adverse effect except for slight drowsiness. The safety of Xe-CT was reconfirmed.

Portal hypertensive symptoms in NASH, such as splenomegaly, hepatic coma, ascites, gastroesophageal varices and other collateral vessels, appear at relatively early stages, and are related to prognosis. We will attempt to predict appearance of portal hypertensive symptoms in NASH and CH-C by hepatic TBF using Xe-CT. Evaluating hepatic TBF of the entire liver is, thus, important. Xe-CT allows repeated noninvasive measurement of hepatic TBF and is useful to elucidate the state of chronic liver disease. In the future, we will attempt to evaluate the difference of local hepatic tissue blood flow such as segments or lobes, so that we hope we will apply the evaluation of before and after treatment such as sclerotherapy (e.g., endoscopic injection sclerotherapy; EIS and balloon-occluded retrograde transvenous obliteration; B-RTO) to varices, antiviral therapy to CH-C, and diet therapy in chronic liver disease. We will attempt to assess correlation between liver function and hepatic TBF in chronic liver disease. Furthermore, the evaluation of hepatic TBF using Xe-CT provides very important information of, not only hepatectomy, but also liver transplantation. Xe-CT can assess, noninvasively and accurately, hepatic TBF and steatosis in the whole liver. We are quite sure that Xe-CT is useful not only in the field of internal medicine but also in that of surgery.

## 4. Material and Methods

### 4.1. Patients

This study included a total of 202 patients with NAFLD and CH-C, who had undergone Xe-CT at the St. Marianna University School of Medicine Hospital (Kawasaki, Japan) between October, 2000, and January, 2013. Liver biopsy was performed for 84 of 93 NAFLD patients and for 90 of 109 CH-C patients. During a 3-day hospitalization examination, we performed Xe-CT before or after each liver biopsy. The NAFLD patients consisted of 58 men and 35 women with a mean age of 52.3 ± 16.0 years and a mean body mass index (BMI) of 28.7 ± 4.5 kg/m^2^. The CH-C patients consisted of 55 men and 54 women, with a mean age of 58.9 ± 10.9 years and a mean BMI of 23.7 ± 3.6 kg/m^2^ (Table 4).

The diagnosis of NAFLD was based on: (1) ethanol intake of <20 g/day; (2) liver biopsy findings showing NAFLD characteristics (e.g., large-droplet fat deposits, hepatocyte ballooning, inflammatory cell infiltration, and fibrosis around the central vein); and (3) the exclusion of other liver diseases, such as viral hepatitis, autoimmune liver disease, and drug-induced liver injury. Diagnosis of CH-C was made by positive anti-HCV antibody and HCV-RNA testing.

Liver biopsy was performed through the right intercostal space under ultrasonographic guidance, using a 16-gauge needle biopsy kit (Quick-Core^®^ biopsy needle set; Cook Medical, Bloomington, IN, USA). Histological diagnosis was confirmed by two experienced pathologists who were blinded to the clinical data. There were 12 patients with Simple Steatosis (SS), who had no fibrosis and inflammatory cell infiltration. The NASH patients were evaluated on the basis of Brunt’s classification [35], and the HCV patients were evaluated on the basis of Desmet’s classification [36].

Subjects were classified into one of three groups, based on fibrosis stage: group 1, no bridging fibrosis; group 2, bridging fibrosis; and group 3, liver cirrhosis. Group 1 included Brunt’s classification stage 1 and 2 and Desmet’s classification stage 1. Group 2 included Brunt’s classification stage 3 and Desmet’s classification stage 2 and 3. Group 3 included Brunt’s classification stage 4 and Desmet’s classification stage 4 (Figure 3).

### 4.2. Imaging

**Figure 4.**
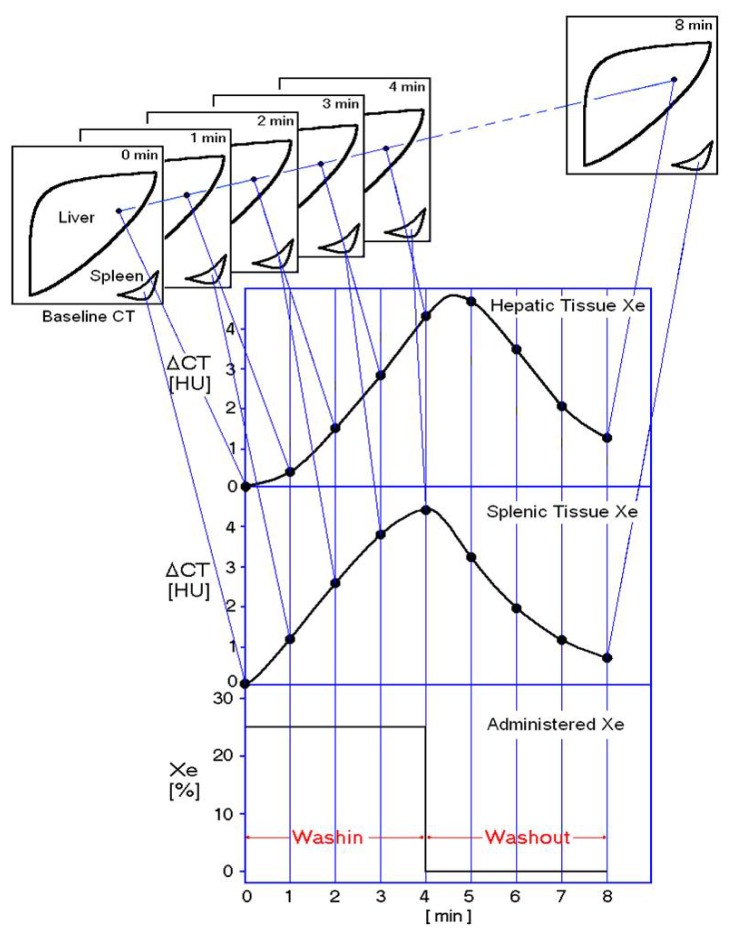
Measuring methods of hepatic TBFs using Xe-CT. Xenon concentration in inhaled gas was 25%, and a 4-min wash-in and 4-min wash-out were used. CT at each of the four levels was performed eight times at 1-min intervals. Patients held their breath during each scan to prevent movement of the liver due to respiration. CT of the spleen was used to measure arterial xenon concentrations.

**Figure 5.**
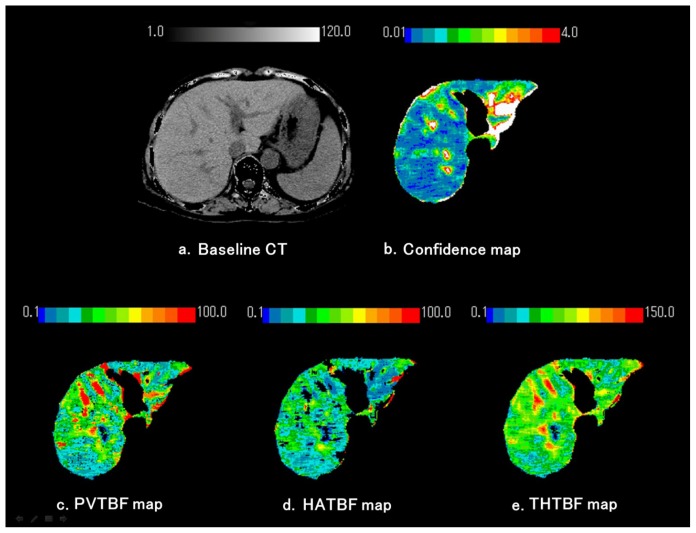
Measurement of hepatic tissue blood flows (TBFs) and confidence values obtained using xenon computed tomography (Xe-CT). Maps were created for portal venous TBF (PVTBF; c), hepatic arterial TBF (HATBF; d), the Xe solubility coefficient, and confidence values for each pixel in the liver, on the basis of changes over time in the Xe-CT numbers in hepatic tissue and spleen. (**a**) Baseline CT; (**b**) Confidence map. The original blood flow maps were modified by automatically excluding any pixels with confidence values exceeding the threshold in the confidence map. The white areas on the confidence map indicate regions of low reliability and were automatically excluded. Confidence values indicate the difference between theoretical and actual changes over time on Xe-CT; (**c**) Portal tissue blood flow (PVTBF) map; (**d**) Hepatic arterial tissue blood flow (HATBF) map; (**e**) Total hepatic tissue blood flow (THTBF) map.

As described in previous publications, we used 25% stable Xe gas in conjunction with an AZ-726 Xe gas inhalation system (Anzai Medical, Tokyo, Japan) [37,38]. The wash-in and wash-out periods were both 4 min (Figure 4). The entire liver was CT-scanned at 1-min intervals at four levels, including the porta hepatis (nine scans in total, including the baseline scan). Using an AZ-7000W image processing system (Anzai Medical, Tokyo, Japan), PVTBF and HATBF were calculated, and PVTBF and HATBF maps were created. THTBF was calculated as the sum of PVTBF and HATBF, and THTBF maps were also created. The time course change rate for the arterial Xe concentration, which was needed to calculate PVTBF and HATBF, was derived using the time course of the Xe concentration in spleen tissue. An Aquilion CT scanner (Toshiba Medical Systems, Tokyo, Japan) was used, with exposure factors of 120 kV, 150 mA, and 13.8 mGy. The TBF maps derived from the Xe-CT scans are shown in Figure 5. All examinations were performed with the patients in the fasting state. Informed consent was obtained from each patient. All study protocols were reviewed and approved by the ethics committee of St. Marianna University Hospital (approval No. 480) (Kawasaki, Japan).

### 4.3. Statistical Analysis

The blood flow in each stage was analyzed by one-way analysis of variance (Tukey’s multiple comparison). Correlation of TBF with progression of fibrosis was analyzed by Spearman’s rank correlation coefficient.

## 5. Conclusions

PVTBF and THTBF decreased due to fat infiltration. Therefore, the hemodynamic changes in NAFLD occur at a relatively earlier stage than that in CH-C. Patients with NASH should be monitored carefully for portal hypertensive complications in the early fibrosis stage.

## Figures and Tables

**Table 1.** Correlation between disease progression (degree of fibrosis) and hepatic TBF in NAFLD.

	SS (*n* = 12)	Stage 1 (*n* = 27)	Stage 2 (*n* = 24)	Stage 3 (*n* = 14)	Stage 4A (*n* = 11)	Stage 4B (*n* = 5)
**PVTBF**	41.83 ± 6.33	34.54 ± 7.57	33.57 ± 7.57	29.99 ± 6.91	28.88 ± 5.73	22.36 ± 4.44
**HATBF**	25.48 ± 8.97	23.10 ± 11.14	17.47 ± 7.28	20.58 ± 8.61	18.99 ± 7.26	14.02 ± 4.88
**THTBF**	67.31 ± 13.03	57.64 ± 14.11	51.04 ± 12.57	50.56 ± 13.86	47.87 ± 9.75	36.38 ± 8.85

**Table 2.** Correlation between disease progression (degree of fibrosis) and hepatic TBF in CH-C.

	Stage 1 (*n* = 34)	Stage 2 (*n* = 29)	Stage 3 (*n* = 21)	Stage 4A (*n* = 14)	Stage 4B (*n* = 11)
**PVTBF**	52.56 ± 13.55	41.70 ± 12.91	39.61 ± 8.79	34.39 ± 9.33	29.86 ± 6.46
**HATBF**	26.82 ± 15.65	22.36 ± 8.14	19.84 ± 11.89	18.67 ± 9.57	20.26 ± 17.79
**THTBF**	79.38 ± 22.88	64.07 ± 16.14	59.45 ± 13.22	53.06 ± 11.89	50.12 ± 17.00

**Table 3.** Comparison of hepatic TBF between NASH and CH-C.

	NASH (*n* = 72)		CH-C (*n* = 90)	*p* value
	*n* = 51	Group 1	*n* = 34	
		
**PVTBF**	34.08 ± 7.51		52.56 ± 13.55	*p* < 0.001
**HATBF**	20.45 ± 9.85		26.82 ± 15.65	0.024
**THTBF**	54.53 ± 13.69		79.38 ± 22.88	*p* < 0.001
	*n* = 14	Group 2	*n* = 50	
		
**PVTBF**	29.99 ± 6.91		40.85 ± 11.35	*p* < 0.001
**HATBF**	20.58 ± 8.61		21.33 ± 9.81	0.796
**THTBF**	50.56 ± 13.86		62.18 ± 15.05	0.012
	*n* = 7	Group 3	*n* = 6	
		
**PVTBF**	28.74 ± 6.78		36.93 ± 8.50	0.079
**HATBF**	15.63 ± 4.04		16.83 ± 4.65	0.627
**THTBF**	44.37 ± 8.01		53.77 ± 10.10	0.088

CT findings were correlated with fibrosis classifications based on liver biopsies, which have been taken from all patients. NASH patients were evaluated on the basis of Brunt’s classification, and CH-C patients were evaluated on the basis of Desmet’s classification. Group 1: non-bridging fibrosis (NASH stage 1 and 2, CH-C stage 1); Group 2: bridging fibrosis (NASH stage 3, CH-C stage 2 and 3); Group 3: liver cirrhosis (NASH stage 4, CH-C stage 4).

**Table 4.** Patient characteristics.

	NAFLD	CH-C
**Number of cases**	93	109
**Gender (Male/Female)**	58/35	55/54
**Age (years)**	52.3 ± 16.0	58.9 ± 10.9
**BMI (kg/m^2^)**	28.7 ± 4.5 ※	23.7 ± 3.6
**Staging for fibrosis**	12/27/24/14/11/5	34/29/21/14/11
	SS/Stage 1/2/3/4A/4B (Brunt’s classification )	Stage 1/2/3/4A/4B (Desmet’s classification )

SS, Simple Steatosis; BMI, Body Mass Index;

※ *p* < 0.001 (unpaired *t*-test).

## Abbreviations

Xe-CT: Xenon computed tomography
TBF: Tissue blood flow
NAFLD: Nonalcoholic fatty liver disease
SS: Simple Steatosis
CH-C: Chronic hepatitis C
US: Ultrasonography
CT: Computed tomography
HATBF: Hepatic arterial tissue blood flow
PVTBF: Portal venous tissue blood flow
THTBF: Total hepatic tissue blood flow
LC: Liver cirrhosis
NASH: Nonalcoholic steatohepatitis

## References

[1] Annet L., Materne R., Danse E., Jamart J., Horsmans Y., van Beers B.E. (2003). Hepatic flow parameters measured with MR imaging and Doppler US: Correlations with degree of cirrhosis and portal hypertension. Radiology.

[2] Alpay H., Birsen S.C., Çetin C., Sener C. (2005). Value of Doppler sonography in assessing the progression of chronic viral hepatitis and in the diagnosis and grading of cirrhosis. J. Ultrasound Med.

[3] Ulusan S., Yakar T., Koc Z. (2011). Evaluation of portal venous velocity with doppler ultrasound in patients with nonalcoholic fatty liver disease. Korean J. Radiol.

[4] Ridolfi F., Abbattista T., Busilacchi P., Brunelli E. (2012). Contrast-enhanced ultrasound evaluation of hepatic microvascular changes in liver diseases. World J. Gastroenterol.

[5] Ronot M., Asselah T., Paradis V., Michoux N., Dorvillius M., Baron G., Marcellin P., van Beers B.E., Vilgrain V. (2010). Liver fibrosis in chronic hepatitis C virus infection: Differentiating minimal from intermediate fibrosis with perfusion CT. Radiology.

[6] Rold an-Alzate A., Frydrychowicz A., Niespodzany E., Landgraf B., Johnson M.K., Wieben O., Reeder S.B. (2013). *In vivo* validation of 4D flow MRI for assessing the hemodynamics of portal hypertension. Magn. Reson. Imaging.

[7] Chiandussi L., Greco F., Sardi G., Vaccarino A., Ferraris C.M., Curti B. (1968). Estimation of hepatic arterial and portal venous blood flow by direct catheterization of the vena porta through the umbilical cord in man. Preliminary results. Acta Hepatosplenol.

[8] Polasek M., Fuchs C.B., Uppal R., Schühle T.D., Alford J.K., Loving S.G., Yamada S., Wei L., Lauwers G.Y., Guimaraes A.R. (2012). Molecular MR imaging of liver fibrosis: A feasibility study using rat and mouse models. J. Hepatol.

[9] Ehling J., Bartneck M., Fech V., Butzbach B., Cesati R., Botnar R., Lammers T., Tacke F. (2013). Elastin-based molecular MRI of liver fibrosis. Hepatology.

[10] Johnson D.W., Stringer W.A., Marks M.P., Yonas H., Good W.F., Gur D. (1991). Stable xenon CT cerebral blood flow imaging: Rationale for and role in clinical decision making. Am. J. Neuroradiol.

[11] Gur D., Good W.F., Wolfson S.K., Yonas H., Shabason L. (1982). *In vivo* mapping of local cerebral blood flow by xenonenhanced computed tomography. Science.

[12] McCuskey R.S., Ito Y., Robertson G.R., McCuskey M.K., Perry M., Farrell G.C. (2004). Hepatic microvascular dysfunction during evolution of dietary steatohepatitis in mice. Hepatology.

[13] Schneider A.R.J., Teuber G., Kriener S., Caspary W.F. (2005). Noninvasive assessment of liver steatosis, fibrosis and inflammation in chronic hepatitis C virus infection. Liver Int.

[14] Lutz H.H., Gassler N., Tischendorf F.W., Trautwein C., Tischendorf J.J. (2012). Doppler ultrasound of hepatic blood flow for noninvasive evaluation of liver fibrosis compared with liver biopsy and transient elastography. Dig. Dis. Sci.

[15] Ikeda H., Suzuki M., Kobayashi M., Takahashi H., Matsumoto N., Maeyama S., Iino S., Sase S., Itoh F. (2007). Xenon computed tomography shows hemodynamic change during the progression of chronic hepatitis C. Hepatol. Res.

[16] Kobayashi M., Suzuki M., Ikeda H., Takahashi H., Matsumoto N., Maeyama S., Sase S., Iino S., Itoh F. (2009). Assessment of hepatic steatosis and hepatic tissue blood flow by xenon computed tomography in nonalcoholic steatohepatitis. Hepatol. Res.

[17] Shigefuku R., Takahashi H., Kobayashi M., Ikeda H., Matsunaga K., Okuse C., Matsumoto N., Maeyama S., Sase S., Suzuki M. (2012). Pathophysiological analysis of nonalcoholic fatty liver disease by evaluation of fatty liver changes and blood flow using xenon computed tomography: Can early-stage nonalcoholic steatohepatitis be distinguished from simple steatosis?. J. Gastroenterol.

[18] Takahashi H., Suzuki M., Ikeda H., Kobayashi M., Sase S., Yotsuyanagi H., Maeyama S., Iino S., Itoh F. (2010). Evaluation of quantitative portal venous, hepatic arterial, and total hepatic tissue blood flow using xenon CT in alcoholic liver cirrhosis—Comparison with liver cirrhosis related to hepatitis C virus and nonalcoholic steatohepatitis. Alcohol. Clin. Exp. Res.

[19] Takahashi H., Suzuki M., Ikeda H., Kobayashi M., Sase S., Yotsuyanagi H., Maeyama S., Iino S., Itoh F. (2007). Evaluation of quantitative portal venous, hepatic arterial, and total hepatic tissue blood flow using xenon CT in alcoholic liver cirrhosis: Comparison with liver cirrhosis C. Alcohol. Clin. Exp. Res.

[20] Takahashi H., Suzuki M., Shigefuku R., Okano M., Hiraishi T., Takagi R., Noguchi Y., Hattori N., Hatsugai M., Nakahara K. (2013). Xenon computed tomography can evaluate the improvement of hepatic hemodynamics before and after endoscopic injection sclerotherapy. J. Gastroenterol.

[21] Flavia D.M., Suzuki A., Sanderson S.O., Lindor K.D., Angulo P. (2012). Prevalence and indicators of portal hypertension in patients with nonalcoholic fatty liver disease. Clin. Gastroenterol. Hepatol.

[22] Seifalian A.M., Mallet S.V., Rolles K., Davidson B.R. (1997). Hepatic microcirculation during human orthotopic liver transplantation. Br. J. Surg.

[23] Seifalian A.M., Chidambaram V., Rolles K., Davidson B.R. (1998). *In vivo* demonstration of impaired microcirculation in steatotic human liver grafts. Liver Transpl. Surg.

[24] Seifalian A.M., Piasecki C., Agarwal A., Davidson B.R. (1999). The effect of graded steatosis on flow in the hepatic parenchymal microcirculation. Transplantation.

[25] Samia I., Wenxuan Y., Winslet M.C., Alexander M., Seifalian A.M. (2003). Impairment of hepatic microcirculation in fatty liver. Microcirculation.

[26] Hayashi N., Kasahara A., Kurosawa K., Sasaki Y., Fusamoto H., Sato N., Kamada T. (1985). Oxygen supply to the liver in patients with alcoholic liver disease assessed by organ-reflectance spectrophotometry. Gastroenterology.

[27] Andersen A.M., Ladefoged J. (1967). Partition coefficient of 133-xenon between various tissues and blood *in vivo*. Scand. J. Clin. Lab. Investig..

[28] Steward A., Allott P.R., Cowles A.L., Mapleson W.W. (1973). Solubility coefficients for inhaled anaesthetics for water, oil and biological media. Br. J. Anaesth.

[29] Gulberg V., Haag K., Rossle M., Gerbes A.L. (2002). Hepatic arterial buffer response in patients with advanced cirrhosis. Hepatology.

[30] Richter S., Mucke I., Menger M.D., Vollmar B. (2000). Impact of intrinsic blood flow regulation in cirrhosis: Maintenance of hepatic arterial buffer response. Am. J. Physiol. Gastrointest. Liver Physiol.

[31] Pasarin M., Abraldes J.G., Rodriguez-Vilarrupla A., La Mura V., Garcia-Pagan J.C., Bosch J. (2011). Insulin resistance and liver microcirculation in a rat model of early NAFLD. J. Hepatol.

[32] Mitsuoka H., Suzuki S., Sakaguchi T., Baba S., Miwa M., Konno H., Nakamura S. (1999). Contribution of endothelin-1 to microcirculatory impairment in total hepatic ischemia and reperfusion injury. Transplantation.

[33] Katagiri H., Ito Y., Ito S., Murata T., Yukihiko S., Narumiya S., Watanabe M., Majima M. (2008). TNF-alpha induces thromboxane receptor signaling-dependent microcirculatory dysfunction in mouse liver. Shock.

[34] Vollmar B., Menger M.D. (2009). The hepatic microcirculation: Mechanistic contributions and therapeutic targets in liver injury and repair. Physiol. Rev.

[35] Brunt E.M., Janney C.G., di Bisceglie A.M., Neuschwander-Tetri B.A., Bacon B.R. (1999). Non-alcoholic steatohepatitis: A proposal for grading and staging the histological lesions. Am. J. Gastroenterol.

[36] Desmet V.J., Gerber M., Hoofnagle J.H., Manns M., Scheuer P.J. (1994). Classification of chronic hepatitis: Diagnosis, grading and staging. Hepatology.

[37] Sase S., Monden M., Oka H., Dono K., Fukuta T., Shibata I. (2002). Hepatic blood flow measurements with arterial and portal blood flow mapping in the human liver by means of xenon CT. J. Comput. Assist. Tomogr.

[38] Sase S., Takahashi H., Ikeda H., Kobayashi M., Matsumoto N., Suzuki M. (2008). Determination of time-course change rate for arterial xenon using the time course of tissue xenon concentration in xenonenhanced computed tomography. Med. Phys.

